# Thyroid hormone enhanced human hepatoma cell motility involves brain-specific serine protease 4 activation via ERK signaling

**DOI:** 10.1186/1476-4598-13-162

**Published:** 2014-07-01

**Authors:** Cheng-Yi Chen, I-Hsiao Chung, Ming-Ming Tsai, Yi-Hsin Tseng, Hsiang-Cheng Chi, Chung-Ying Tsai, Yang-Hsiang Lin, You-Ching Wang, Chie-Pein Chen, Tzu-I Wu, Chau-Ting Yeh, Dar-In Tai, Kwang-Huei Lin

**Affiliations:** 1 Department of Biochemistry, School of Medicine, Chang-Gung University, 259 Wen-hwa 1 Road, Taoyuan, Taiwan; 2 Department of Medical Research, Mackay Memorial Hospital, 251 Taipei, Taiwan; 3 Department of Nursing, Chang-Gung University of Science and Technology, 333 Taoyuan, Taiwan; 4 Medical Research Central, Chang Gung Memorial Hospital, 333 Taoyuan, Taiwan; 5 Division of High Risk Pregnancy, Mackay Memorial Hospital, 104 Taipei, Taiwan

**Keywords:** Thyroid hormone receptor, Secreted protein, BSSP4, SILAC, ERK 1/2-C/EBPβ-VEGF, Mmotility

## Abstract

**Background:**

The thyroid hormone, 3, 3′, 5-triiodo-L-thyronine (T_3_), has been shown to modulate cellular processes via interactions with thyroid hormone receptors (TRs), but the secretory proteins that are regulated to exert these effects remain to be characterized. Brain-specific serine protease 4 (BSSP4), a member of the human serine protease family, participates in extracellular matrix remodeling. However, the physiological role and underlying mechanism of T_3_-mediated regulation of BSSP4 in hepatocellular carcinogenesis are yet to be established.

**Methods:**

The thyroid hormone response element was identified by reporter and chromatin immunoprecipitation assays. The cell motility was analyzed via transwell and SCID mice. The BSSP4 expression in clinical specimens was examined by Western blot and quantitative reverse transcription polymerase chain reaction.

**Results:**

Upregulation of BSSP4 at mRNA and protein levels after T_3_ stimulation is a time- and dose-dependent manner in hepatoma cell lines. Additionally, the regulatory region of the BSSP4 promoter stimulated by T_3_ was identified at positions -609/-594. BSSP4 overexpression enhanced tumor cell migration and invasion, both in vitro and in vivo. Subsequently, BSSP4-induced migration occurs through the ERK 1/2-C/EBPβ-VEGF cascade, similar to that observed in HepG2-TRα1 and J7-TRα1 cells. BSSP4 was overexpressed in clinical hepatocellular carcinoma (HCC) patients, compared with normal subjects, and positively associated with TRα1 and VEGF to a significant extent. Importantly, a mild association between BSSP4 expression and distant metastasis was observed.

**Conclusions:**

Our findings collectively support a potential role of T_3_ in cancer cell progression through regulation of the BSSP4 protease via the ERK 1/2-C/EBPβ-VEGF cascade. BSSP4 may thus be effectively utilized as a novel marker and anti-cancer therapeutic target in HCC.

## Introduction

The thyroid hormone (TH) acts as a pleiotropic regulator in growth, differentiation, proliferation and other physiological processes by interacting with thyroid hormone response elements (TREs) located in the regulatory regions of target genes [1]. Indeed, TH is required to maintain the metabolic rate and oxygen consumption in almost all tissues [2]. TRs interact with the retinoid X receptor (RXR) to form heterodimers that influence target genes by binding to their TRE regions [3]. Two TR genes, TRα and TRβ, have been identified on human chromosomes 17 and 3, respectively. Alternative splicing and promoter usage of primary transcripts have been shown to generate TRα1, TRα2, TRβ1 and TRβ2 receptor isoforms [4].

Previous microarray experiments by our group demonstrated that numerous genes encoding coagulation factor system components, plasma proteins, nuclear receptor coactivator, anti-metastatic proteins, proteases and oncogenes are regulated by T_3_[2]. Recently, we employed stable isotope labeling with amino acids in cell culture (SILAC)-based quantitative proteomic approaches, with a view to systematically investigating the T_3_-regulated secretome [5]. Consequently, several secreted proteins and the urokinase plasminogen activator (uPA) system were identified and quantified, clearly indicative of a role of T_3_ in the modulation of secretory proteins. Earlier proteomic analyses further revealed a significant increase in brain-specific serine protease 4 (BSSP4) involved in the uPA system [6], quantified in one of the three SILAC experiments (T_3_/Td >3), in T_3_-treated HepG2-TRα1 cells.

BSSP4 is a member of the human chromosome 16p13.3 serine protease family. The protease is also known as tryptase ϵ and protease serine S1 family member 22 [7]. Previous study by Yasuda *et al*. [6] reported that BSSP4 catalyzes the progression of zymogen pro-uPA to active uPA. The uPA system, a serine protease family comprising tissue-type plasminogen activator (tPA), uPA, uPA receptor (uPAR) and plasminogen activator inhibitors (PAIs), is universally found in almost all cancer types [8]. While BSSP4 has been shown to play a role in different cancers [9], few studies to date have focused on its specific functions in cancer biology.

In the current study, we investigated T_3_-mediated regulation of BSSP4 and its underlying physiological significance in hepatoma cell lines. Our results indicate that T_3_-regulated BSSP4 induces cancer progression via the ERK1/2-C/EBPβ-VEGF cascade in hepatoma cells. Moreover, BSSP4 is consistently overexpressed in HCC patients, compared with normal subjects, supporting its utility as a novel marker.

## Results

### Effects of T_3_ on BSSP4 mRNA and protein expression

**Figure 1:**
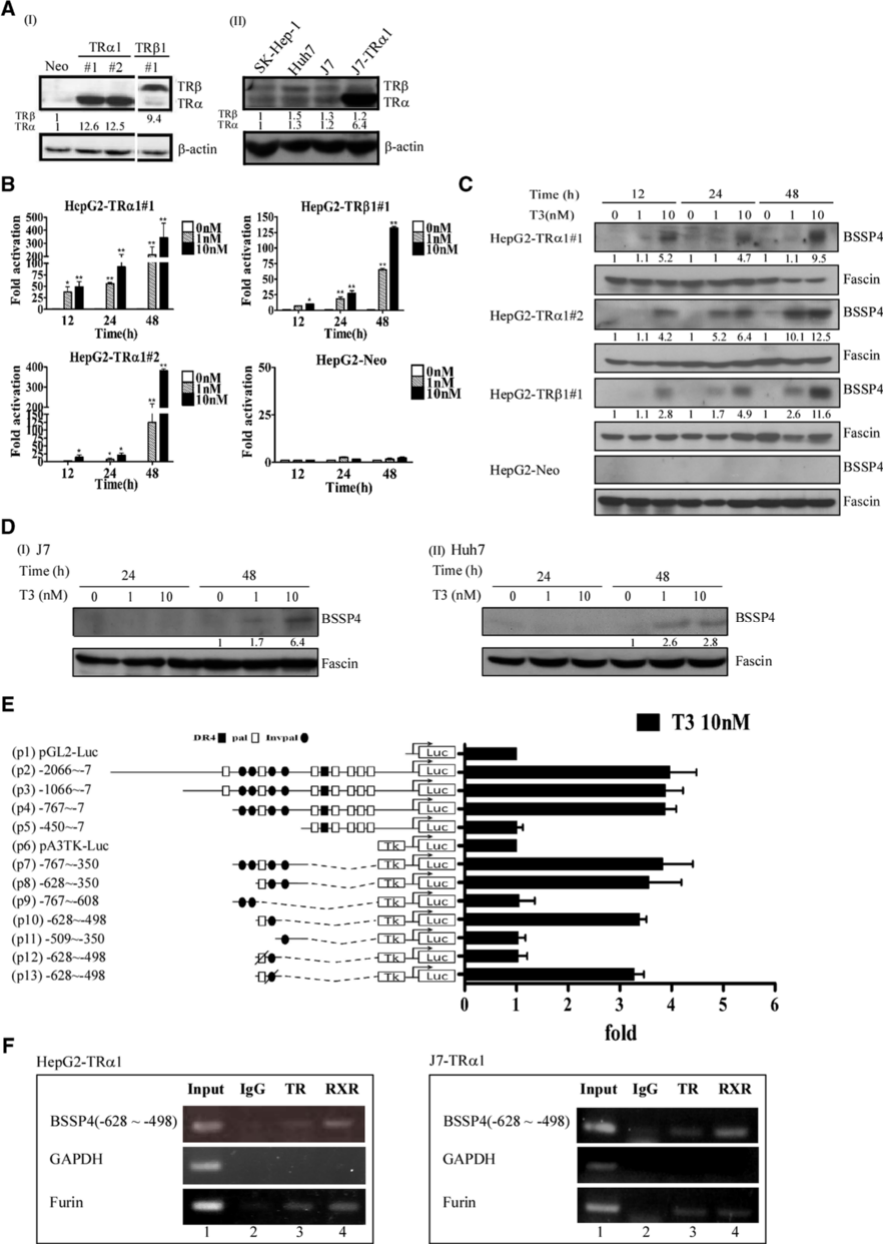
**T**_**3**_-**regulates BSSP4 mRNA and protein expression in HepG2 cells. (A)** Expression of TR in cell extracts of overexpressed-TR and parental cell lines was determined via Western blotting. The positions of 47 kDa TRα1 and 55 kDa TRβ1 are indicated. BSSP4 expression was determined in the three stable HepG2-TR lines and HepG2-neo cells at 12–48 h in the absence or presence of 1 and 10 nM T_3_ using **(B)** Q-PCR and **(C)** Western blotting. **(D)** T_3_ stimulated BSSP4 expression in J7 and Huh7 cells expressing endogenous TR was determined by Western blotting. **(E)** HepG2-TRα1 cells were transfected with the luciferase reporter plasmid driven by the *BSSP4* 5′-flanking region (positions -2066 to -7 containing twelve putative TRE sites) with or without pA3TK-luc. Promoter activities were calculated, relative to 0 nM T_3_ (+T_3_/-T_3_), and further normalized to the pA3TK-luc control as well as β-galactosidase activity (T_3_-induced changes were normalized to that of β-gal). Columns, mean values obtained from at least three independent experiments performed in triplicate; bars, SE. **(F)** ChIP assay demonstrating that TR is recruited to the *BSSP4* 5′-flanking region, together with RXR in HepG2-TRα1 and J7-TRα1 cells. Two sets of primers for *BSSP4* TRE, positive control TRE (*FURIN*) and negative control (*GAPDH*) were prepared.

Several isogenic HepG2 cell lines stably expressing high levels of wild-type TRα1 and TRβ1 (HepG2-TRα1 and HepG2-TRβ1, respectively) were established. Specifically, four HepG2 cell lines, HepG2-TRα1#1, HepG2-TRα1#2, HepG2-TRβ1#1 and HepG2-Neo, and J7-TRα1, and parental cells expressing various levels of TR (Figure 1A) were used for analyses. Notably, BSSP4 expression was stimulated by T_3_ in the HepG2-TRα1#1, HepG2-TRα1#2 and HepG2-TRβ1 cell lines at both the mRNA (Figure 1B) and protein levels (Figure 1C) in a time- and dose-dependent manner. In contrast, T_3_ had a marginal or no effect on BSSP4 expression in HepG2-Neo cells (Figure 1B, C). Moreover, we have examined the BSSP4 regulation by T_3_ in parental cells expressing endogenous TR, such as J7 (Figure 1D, I), Huh7 (Figure 1D, II) and SK-Hep-1 cells (Additional file 1: Figure S1B). We have depleted the TRα and TRβ expression with siRNA in SK-Hep1 cell (Additional file 1: Figure S1A). The BSSP4 regulation was abolished significantly after silencing the TRα and TRβ expression (Additional file 1: Figure S1B). Several HCC cell lines expressing low levels of endogenous TR proteins can be induced the BSSP4 expression with a lesser extent after T_3_ application. According to the data, we conclude the BSSP4 regulation by T_3_ can be observed in the parental cells as well as TR overexpression system.

### T_3_ induces BSSP4 expression at the transcriptional level

The reporter assay was performed to determine the position of the thyroid hormone response element (TRE) to further clarify the regulatory effects of T_3_ on *BSSP4* at the transcriptional level. The *BSSP4* 5′-flanking region encompassing nucleotides -2066/-7 (relative to the transcription initiation site) with numerous predicted putative TREs (Figure 1E) was cloned and inserted upstream of the luciferase reporter gene in pGL2-luc (Construct p1) to generate Construct p2. The pA3TK-luc construct containing a minimum thymidine kinase promoter was designated Construct p6. Serial deletion mutants were additionally generated (Figure 1E). The transcriptional activities of the *BSSP4* promoter fragments are illustrated in Figure 1E. Among these, only the p10 construct containing two putative TREs was activated about 3.5-fold by T_3_ in HepG2-TRα1 cells. The two TREs in the p10 fragment were sequentially mutated to yield p12 and p13 constructs. However, upon mutation of the other putative TRE (pal), luciferase activity of the p12 construct was completely abolished (Figure 1E). These findings suggest that T_3_ regulates *BSSP4* at the transcriptional level by binding to the putative TRE site between positions -628/-498 (p10) encompassing a pal-like sequence between positions -609 to -594 (AGGTCCTTGCTGTCCT).

### TR and RXR proteins form a complex with TRE (-609 ~ -594) within the BSSP4 promoter

To further determine whether BSSP4 TRE (pal) is directly targeted by TR proteins, the ChIP assay was performed. TR proteins were clearly associated with the TRE region of the *BSSP4* promoter *in vivo* both HepG2-TRα1 and J7-TRα1 cells (Figure 1F). Notably, TRα1 and RXRα were recruited to the TRE-binding site (Figure 1F, lanes 3, 4), whereas control IgG produced only background levels (Figure 1F, lane 2). Accordingly, we propose that the TRα1 and RXRα complexes bind to the *BSSP4* promoter for transcriptional regulation.

### BSSP4 is associated with cancer progression in vitro

**Figure 2:**
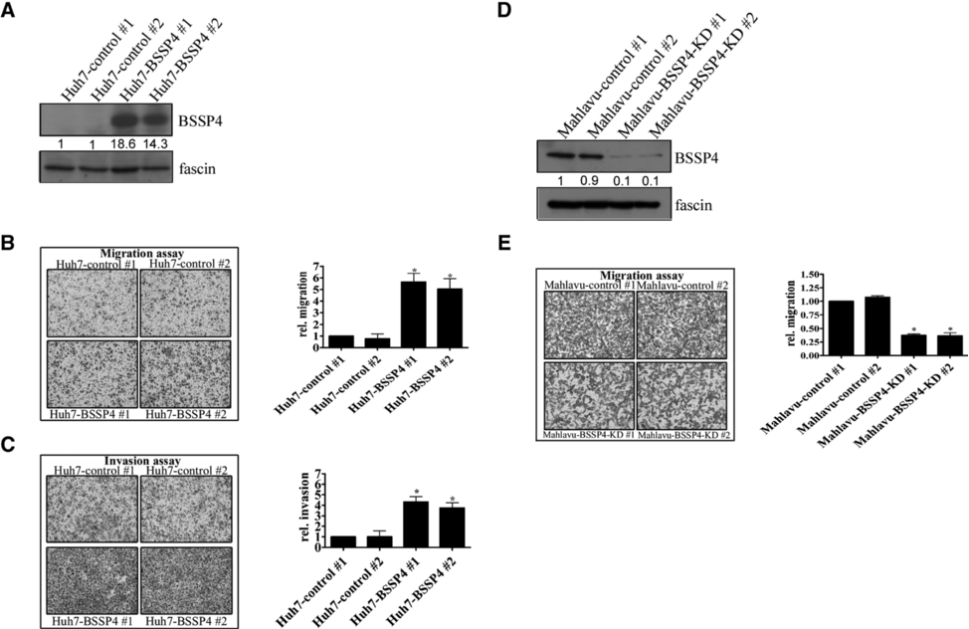
**BSSP4 promotes cell motility *in vitro.*** Expression of BSSP4 was detected in BSSP4-overexpressing clones (#1, #2) and controls (control #1, #2) with Western blotting **(A)**. **(B)** Migration and **(C)** invasion abilities were analyzed in two BSSP4 overexpressing and two control cell lines using a Transwell assay. The number of cells traversing the filter to the lower chamber was expressed as the total number of cells to provide an index of migration and invasion activity. Transwell filters were stained with crystal violet in the left panel, and migration and invasion abilities quantified in the right panel. **(D)** Expression of BSSP4 in Mahlavu-control cells (#1, #2) and Mahlavu-BSSP4-KD cells (#1, #2) was determined by Western blotting. The migration ability was also examined with a Transwell assay **(E)**.

We detected the BSSP4, TRα and TRβ expression in six hepatoma cell lines (Additional file 2: Figure S2). The positive correlation of BSSP4 vs. TRα/TRβ expression was observed in most of the hepatoma cell lines. To determine the specific functions of BSSP4, control cell lines (Figure 2A, Huh7-control #1 and #2) and those overexpressing BSSP4 (Figure 2A, Huh7-BSSP4 #1 and #2) were established. BSSP4 has been detected in several cancer types [9]. The protein was originally identified as a member of the human serine protease family, also designated tryptase ϵ [7]. In view of several earlier findings that proteases function in extracellular matrix remodeling during cancer cell progression and development [10], we aimed to ascertain whether BSSP4 also plays a role in cancer processes.

The proliferation rates of the two stably overexpressing BSSP4 cell lines (Huh7-BSSP4 #1 and #2) were similar to those of the two control cell lines (Huh7-control #1 and #2), as shown in Additional file 3: Figure S3A. However, cell lines overexpressing BSSP4 displayed significantly increased (~3 to 6-fold) migration (Figure 2B) and invasion (Figure 2C), compared with control cells. Our results indicate that BSSP4 functions in cell migration and invasion, but has no effect on cell growth. Furthermore, after depleted BSSP4 in Mahlavu cells, the migration ability of Mahlavu-BSSP4-depleted cells was decreased compared with Mahlavu–control cells by the Transwell assay (Figure 2D, E). Based on the functional assay in BSSP4-depleted and BSSP4-overexpressed cells, BSSP4 has the ability to accelerate tumor cell migration.

### BSSP4 influences EMT progression markers

**Figure 3:**
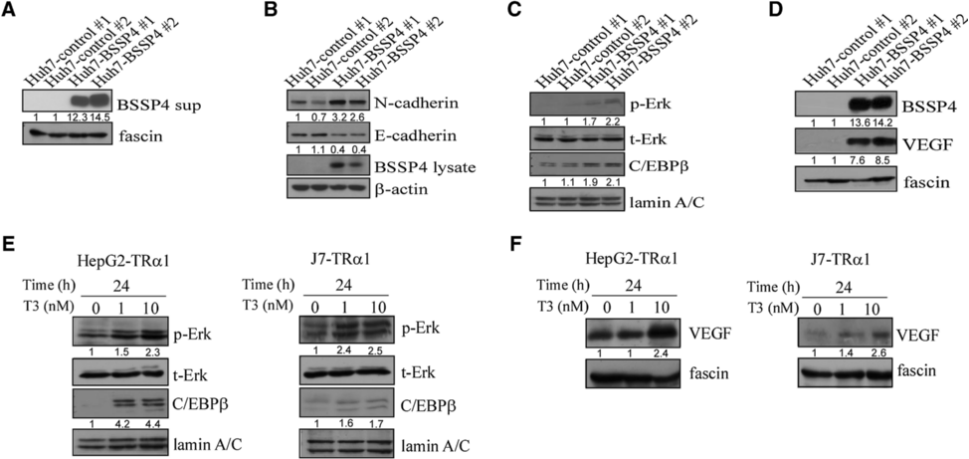
**T**_**3 **_**and BSSP4 enhance the expression of intracellular and extracellular cancer**-**related molecules.** BSSP4-overexpressing clones (Huh7-BSSP4 #1, #2) and controls (Huh7-control #1, #2) were detected via Western blotting **(A)**. Expression of EMT-related markers, such as E-cadherin and N-cadherin, in cell extracts **(B)**, phosphate-ERK and C/EBPβ in the nucleus **(C)**, and VEGF in the conditioned medium **(D)** were examined using Western blotting. Expression patterns of phosphate-ERK and C/EBPβ **(E)** in the nucleus and VEGF in the medium **(F)** in both HepG2-TRα1 and J7-TRα1 cells were established.

To further clarify the mechanisms involved in BSSP4-regulated cell migration and invasion, we selected several markers of epithelial-mesenchymal transition (EMT) for examination [11]. Figure 3A presents BSSP4 expression levels in Huh7-BSSP4 and Huh7-control cells. The N-cadherin level was increased and E-cadherin decreased in BSSP4-overexpressing cells (Huh7-BSSP4 #1 and #2), compared with control cells (Huh7-control #1 and #2), on a Western blot (Figure 3B). Based on these results, we propose that the EMT process is involved in BSSP4-mediated cancer cell progression.

### BSSP4 and T_3_ regulate cancer-related molecules

Previously, Yasuda *et al*. [6] demonstrated that BSSP4 is a serine protease that catalyzes the progression of zymogen pro-uPA to form active uPA. Moreover, the uPA-uPAR system has been linked to the mitogen-activated protein kinase (MAPK)/extracellular signal-regulated kinase (ERK) signaling pathway [12-14]. ERK kinases are frequently abnormally activated in most human cancers, leading to proliferation and acceleration of other oncogenic processes, such as angiogenesis and survival [15]. Accordingly, we examined whether the ERK pathway is implicated in BSSP4-induced phenotypes. Our results showed marked upregulation of phosphate-ERK in BSSP4-overexpressing cells (Huh7-BSSP4 #1 and #2), compared with control cells (Huh7-control #1 and #2) (Figure 3C). A number of groups have identified CCAAT/enhancer-binding protein beta (C/EBPβ and vascular endothelial growth factor (VEGF) as ERK downstream genes [16]. VEGF facilitates the formation of new vessels in angiogenesis and plays a critical role in tumor metastasis. Its expression is highly correlated with tumor cell metastasis potency in various cancers [17]. Similarly, C/EBP plays diverse roles in multiple cellular processes, such as the cell cycle, extracellular signaling, tissue development, and is implicated in cancer processes [18]. Subsequently, we investigated whether C/EBPβ and VEGF are targeted by BSSP4. Notably, C/EBPβ was upregulated in the nucleus and VEGF was extensively secreted into the medium in BSSP4-expressing cells (Huh7-BSSP4 #1 and #2), compared with their control counterparts (Huh7-control #1 and #2) (Figure 3C, D).

We further examined whether this BSSP4-mediated mechanism occurs in T_3_-regulated hepatoma cells. Our results showed upregulation of nuclear phosphate-ERK and C/EBPβ after T_3_ stimulation in both HepG2-TRα1 and J7-TRα1 cells (Figure 3E). Additionally, VEGF secretion into the medium was increased in T_3_-treated HepG2-TRα1 and J7-TRα1 cells (Figure 3F). Accordingly, we conclude that stimulation of ERK, C/EBPβ and VEGF expression by T_3_ in HepG2-TRα1 or J7-TRα1 cells is mediated via BSSP4.

### BSSP4 and T_3_ mediate cell migration via the ERK signaling pathway

**Figure 4:**
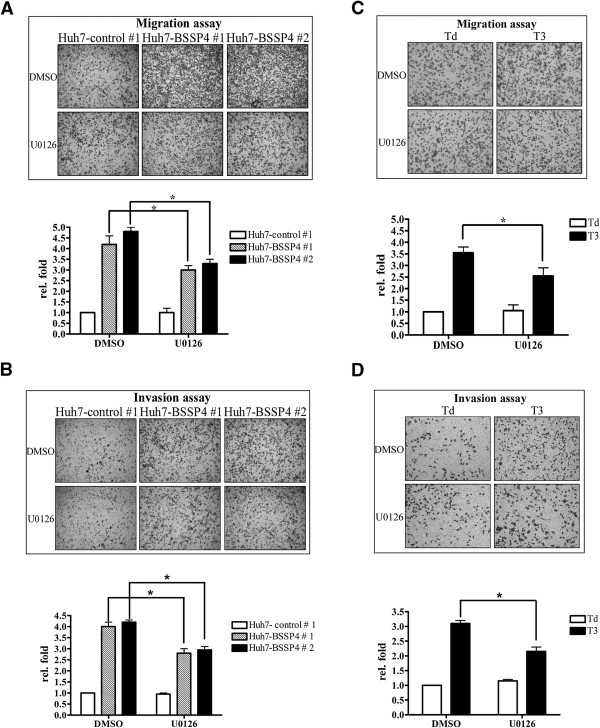
**BSSP4**- **and T**_**3**_-**mediated cell migration occurs via the ERK signaling pathway.** The transwell migration **(A, C)** and invasion assays **(B, D)** were performed for two BSSP4-expressing (Huh7-BSSP4 #1, 2) and one control (Huh7-BSSP4 #1) cell line (A, B), and J7-TRα1 cells **(C, D)**. Stable lines (10^5^) **(A, B)** and T_3_-treated J7-TRα1 (5 × 10^4^) **(C, D)** cells were treated with DMSO and U0126 (10 μM) added to the upper chamber of Transwell units, followed by incubation for 24 h. Transwell filters were stained with crystal violet in the upper panel, and migration ability quantified in the lower panel. Values are shown as fold increase of Huh7-BSSP4 relative to Huh7-control, and T_3_ relative to Td. Differences were analyzed using One-way ANOVA, *P < 0.05.

In view of the above results, we hypothesized that T_3_ regulates BSSP4 through the ERK-C/EBPβ-VEGF cascade, leading to cancer cell progression. To examine this possibility, we treated stable Huh7-BSSP4 cell lines with DMSO and U0126, a MEK inhibitor [19,20], followed by the migration and invasion assays. The migration and invasion ability of BSSP4-overexpressing cells (Huh7-BSSP4 #1 and #2) was increased about 4-fold, relative to control cells (Huh7-control #1) (Figure 4A, B). However, this increase was abolished by approximately 25% upon U0126 treatment of BSSP4-expressing cells (Huh7-BSSP4 #1 and #2) (Figure 4A, B). The quantified results are shown in Figure 4A, B, lower panel. Based on these findings, we propose that the ERK pathway is implicated in BSSP4-mediated cell migration. We further ascertained whether this phenomenon occurs in T_3_-regulated hepatoma cells. As expected, the migration and invasion ability of J7-TRα1 cells was enhanced (~3.5-fold) after T_3_ stimulation (Figure 4C, D), which was attenuated (~30%) upon U0126 treatment (Figure 4C, D). The quantified results are illustrated in Figure 4C, D, lower panel. Thus, BSSP4 appears to mediate cell migration through the ERK signaling pathway.

### The ERK-C/EBPβ-VEGF pathway is involved in BSSP4 and T_3_-mediated cell migration

**Figure 5:**
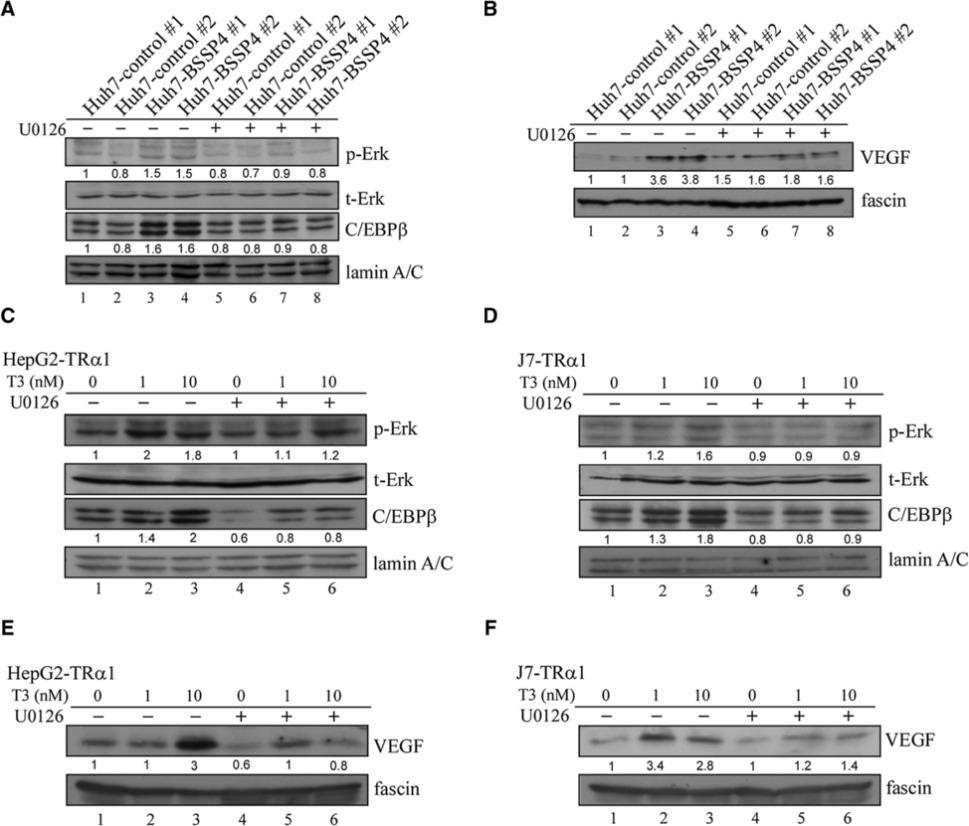
**T**_**3 **_**and BSSP4 promote cell migration via the ERK**-**C**/**EBPβ**-**VEGF cascade.** We determined the expression patterns of phosphate-ERK and C/EBPβ in the nucleus **(A, C, D)** and VEGF in conditioned medium **(B, E, F)** in BSSP4-expressing clones (Huh7-BSSP4 #1, #2), controls (Huh7-control #1, #2) **(A, B)**, and T_3_-stimulated (0, 1, 10 nM) HepG2-TRα1 **(C, E)** and J7-TRα1 **(D, F)** treated with DMSO or U0126 (10 μM) using Western blot analysis.

We observed upregulation of p-ERK, C/EBPβ and VEGF in both BSSP4-overexpressing and T_3_-treated hepatoma cells. Moreover, the ERK signaling pathway is clearly involved in BSSP4- and T_3_-modulated cell migration ability. This led us to investigate whether C/EBPβ and VEGF are influenced by blocking the ERK signaling pathway. Our results showed that expression levels of nuclear p-ERK and C/EBPβ are decreased in stable Huh7-BSSP4 cells after U0126 treatment, compared with those in control cells (Figure 5A, lanes 7, 8 vs 3, 4). VEGF secretion into the medium of stable Huh7-BSSP4 cells was additionally attenuated following U0126 treatment (Figure 5B, lanes 7, 8 vs 3, 4). We further determined whether BSSP4 has a similar influence in T_3_-treated hepatoma cells. Interestingly, in both T_3_-treated HepG2-TRα1 and J7-TRα1 cells, expression of p-ERK and C/EBPβ in the nucleus was blocked after U0126 treatment, compared with control cells (Figure 5C, D). Furthermore, VEGF secretion into the medium was significantly attenuated following U0126 treatment in both T_3_-treated cell lines (Figure 5E, F). Furthermore, the p-VEGFR and VEGFR expression were determined in BSSP4- and TR-overexpressing cells. The p-VEGFR level was increased in BSSP4-overexpressing cells. Consistently, the p-VEGFR expression was up-regulated after T_3_ treatment in the HepG2-TRα cell (Additional file 4: Figure S4). A Similar result can be observed in J7-TRα cell (data not shown). Therefore, we speculate that BSSP4 influenced tumor motility may through VEGF-VEGFR cascade.

The relationship between BSSP4 and cancer-related molecules and pathways was investigated using Human Cancer PathwayFinder RT Profiler metastasis-associated PCR Arrays (Quiagen; Kowloon, Hong Kong). We found 20 genes were altered (about 1.5 ~ 3 fold) in BSSP4 overexpressing cells. The BSSP4-regulated molecules are divided into several categories based on the functions such as cytokines and chemokine pathway (IL-18, CCL7, CXCL12), cell to cell adhesion pathway (PNN, SYK, MCAM), metastasis-related genes (GNRH1, TIMP2, KISS1R, TSHR, TRPM1, SSTR2), transcription factors and regulators (RORB, NR4A3, SMAD2, SMAD4), ECM cleavage pathway (MMP7) and cell cycle regulation (PTEN) were altered (Additional file 5: Figure S5). Thus, BSSP4 may play a role in cancer progression via alteration of related genes. Our results collectively indicate that BSSP4 is upregulated by T_3_, and subsequently activates the ERK-C/EBPβ-VEGF cascade to promote cancer cell progression.

### BSSP4 is associated with cell motility in vitro and in vivo

**Figure 6:**
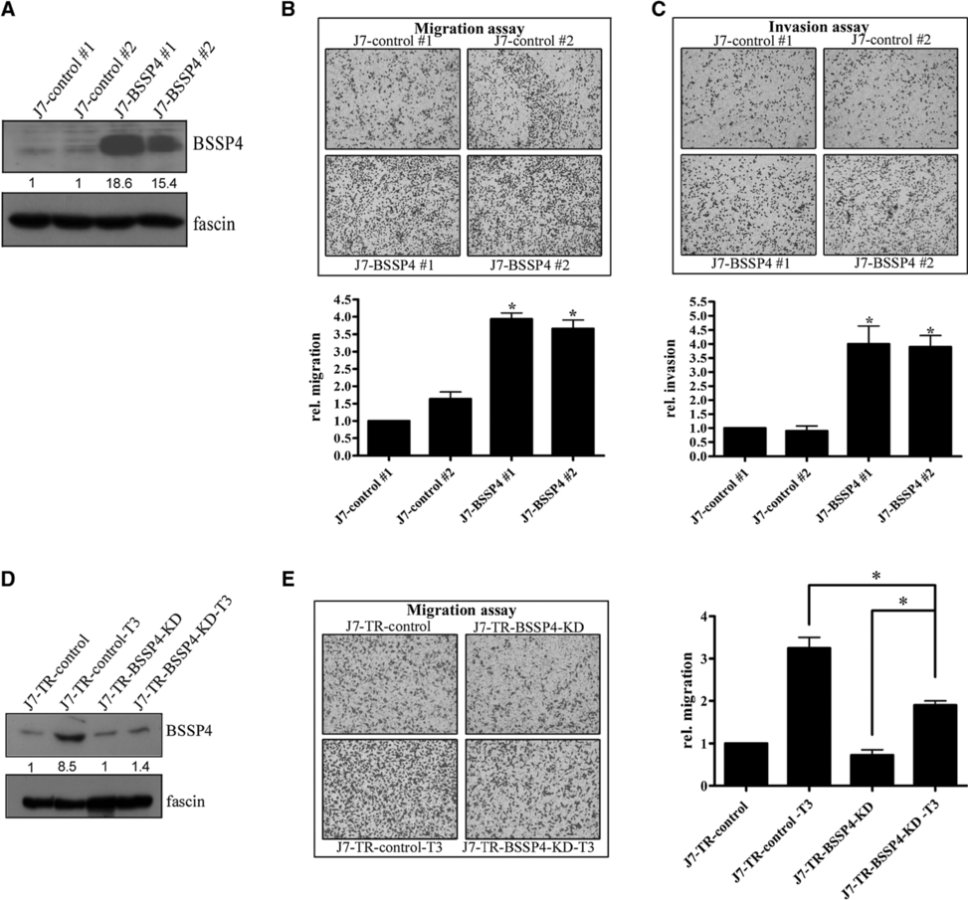
**BSSP4 increases cell motility *in vitro.*** Expression of BSSP4 was detected in BSSP4-expressing clones (#1, #2) and controls (control #1, #2) with Western blotting **(A)**. **(B)** Migration and **(C)** invasion abilities of two BSSP4-overexpressing and two control cell lines were analyzed using a Transwell assay. Transwell filters were stained with crystal violet in the upper panel, and migration and invasion abilities quantified in the lower panel **(B, C)**. The BSSP4 expression in J7-TR-control cells and J7-TR-BSSP4-KD cells treated with/without T_3_ was detected by Western blotting **(D)**. The migration ability was determined with a Transwell assay (**E**, left panel), and the quality index was illustrated in E, right panel. Values are presented as fold increase of J7-BSSP4, relative to J7-control. Differences were analyzed using One-way ANOVA, *P < 0.05.

To verify whether the *in vitro* effect of BSSP4 also occurs *in vivo*, SCID mice were injected with Huh7-BSSP4 and Huh7-control cells. However, no tumors were observed in the lung and liver in either cell line. Based on these findings, we speculated that the Huh7 hepatoma cell line is non-tumorigenic. Therefore, a stable J7-BSSP4 cell line was established. Notably, the BSSP4 level was increased in two stably expressing BSSP4 clones (J7-BSSP4 #1 and #2), compared to two control cell lines (J7-control #1 and #2) (Figure 6A). Similar to Huh-7 cells, proliferation was not influenced in stable BSSP4-overexpressing J7 cell lines, compared with controls (Additional file 3: Figure S3B). Moreover, BSSP4-overexpressing cells (J7-BSSP4 #1 and #2) showed higher migration and invasion abilities, relative to their control cell counterparts (J7-control #1 and #2, respectively) (Figure 6B, C). The *in vitro* phenotypes were similar between Huh7-BSSP4 and J7-BSSP4 cell lines (Figures 2 and 6). Additionally, we have established J7-TR-BSSP4 knockdown (KD) cell lines. The BSSP4 expression in control cells was induced by T_3_, however, the induction was abolished in the J7-TR-BSSP4 KD cells (Figure 6D, E). Obviously, the migration ability of J7-TR-control cells was enhanced by T_3_ stimulation, however, the effect was attenuated in the J7-TR-BSSP4 KD cells (Figure 6E) restoring a more “normal” phenotype. The migration ability was significantly (*p* < 0.018) restored in J7-TR-BSSP4-KD-T_3_ cell line after T3 treatment compared to the BSSP4 silencing J7-TR-BSSP4-KD cell line (Figure 6E, right panel). Based on the evidences, the migration phenotype in the T_3_-treated hepatoma cells can be restored in a BSSP4-KD condition.

**Figure 7:**
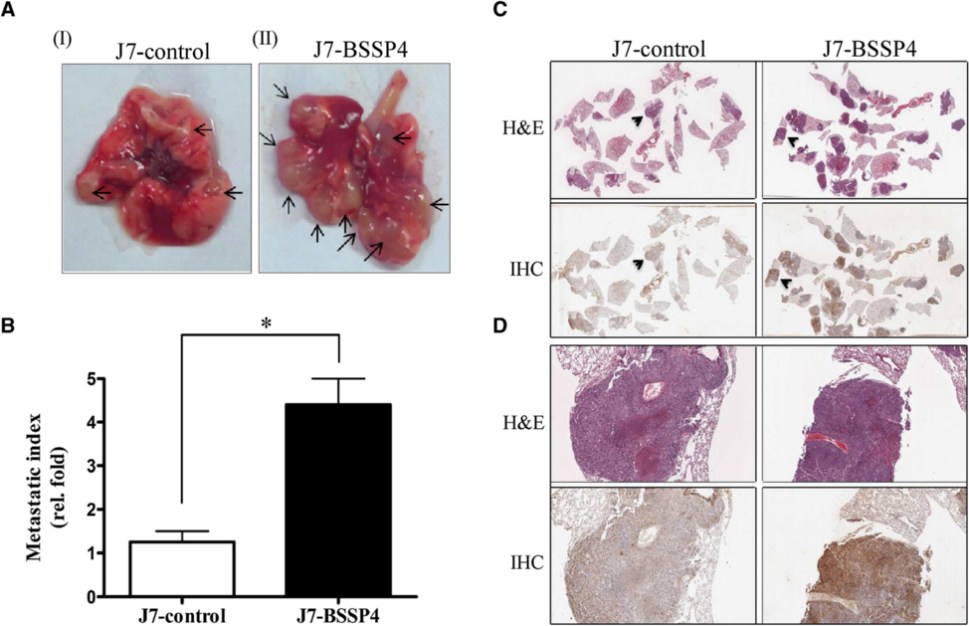
**BSSP4 increases cell motility *in vivo.*** The images **(A)** depict tumor foci of lung sections (arrows indicated) in both J7-control (**A**, I) and J7-BSSP4 cells (**A**, II). The metastatic index (fold, density of tumor numbers in per cm^2^ area) in lung for J7-control and J7-BSSP4 cells is specified **(B)**. Tumor foci and BSSP4 expression of J7-control (**C**, left panel) and J7-BSSP4 cells (**C**, right panel) were examined using H & E staining (**C**, upper panel) or IHC (**C**, lower panel), respectively. The tumor foci covered almost all areas seen in **(D)** (**C** and **D** represent different magnifications, arrowheads in **C** are magnified in **D**). Values are presented as fold increase of J7-BSSP4, relative to J7-control. Differences were analyzed using One-way ANOVA, *P < 0.05.

SCID mice were employed to examine whether the *in vitro* effects of J7-BSSP4 can be replicated in *vivo*. Significantly, in SCID mice, J7-BSSP4 cells formed higher numbers of lung foci (Figure 7A) and had a higher metastatic index (Figure 7B), compared with control cells, as well as elevated BSSP4 expression, as evident from H & E staining (Figure 7C, D, upper panel) and IHC (Figure 7C, D, lower panel) analyses, respectively. Based on these findings, we conclude that BSSP4 promotes cell migration and invasion in J7 hepatoma cells, both *in vitro* and *in vivo*.

### BSSP4 is upregulated in human HCC and animal models

**Figure 8:**
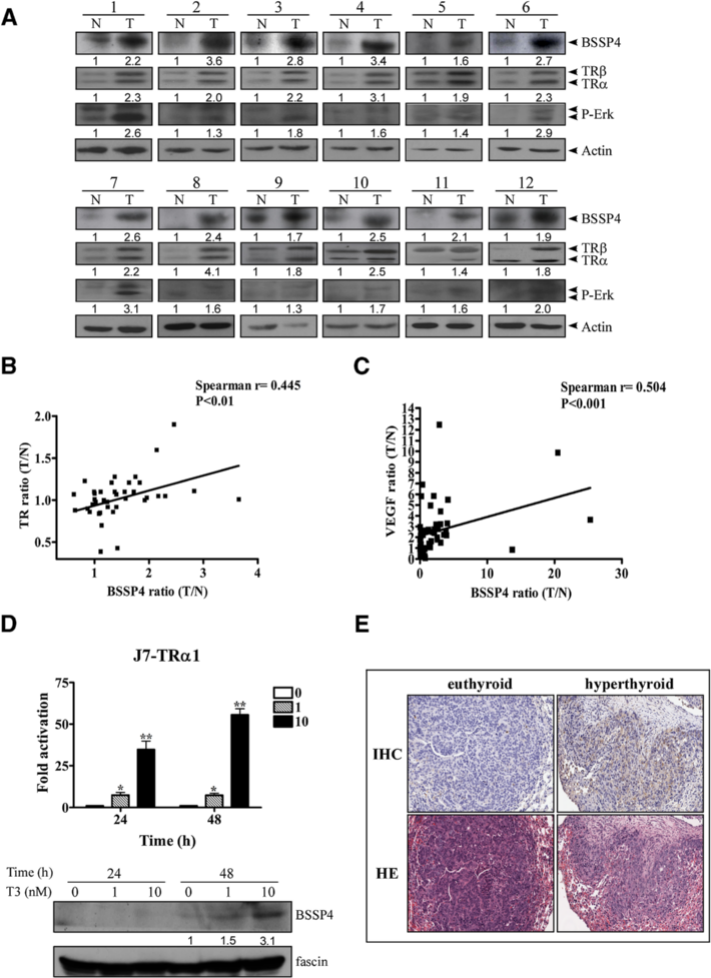
**BSSP4 expression is upregulated *in vitro *and *in vivo***, **and the T/N ratios of BSSP4 and TR levels are positively correlated in HCC. (A)** The BSSP4, TRα/TRβ and P-Erk proteins in HCC specimens were determined using Western blotting. TRs and BSSP4 were overexpressed in 12 representative tumor tissues. The correlations of T/N ratios between BSSP4 and TR **(B)** and BSSP4 and VEGF **(C)** were analyzed using linear regression analysis. BSSP4 regulation by T_3_ at different concentrations (0, 1 and 10 nM) and time-points (24 and 48 h) was examined in J7-TRα1 cells at **(D)** both mRNA (**D**, upper panel) and protein (**D**, lower panel) levels using Q-PCR (**D**, upper panel) and Western blotting (**D**, lower panel), respectively. **(E)** Lung sections of SCID mice injected with J7-TRα1 cells treated with hyper-T_3_ (hyperthyroid) and administered normal drinking water (euthyroid) were analyzed with IHC (upper panel) and H & E (lower panel) staining to examine BSSP4 expression (upper panel) and tumor foci (lower panel).

Next, we examined the clinicopathologic significance of BSSP4 expression in HCC and evaluated the correlation between TRs and BSSP4. Overall, 74 consecutive HCC patients were enrolled, and their BSSP4 levels analyzed using Western blot. Among the 74 HCC sample pairs, the BSSP4 was overexpressed in 52.7% (39 of 74) cancerous tissues, compared with matched noncancerous tissues. Furthermore, both TRα1 and TRβ1 levels were elevated by about 29.7% (22 of 74) in cancerous tissues. We have examined the p-ERK in clinical samples (Figure 8A). The p-ERK expression was higher in tumor than the normal counter- parts, and the tendency was the same with BSSP4 expression. Increased BSSP4 expression and concomitantly elevated TR levels in HCC tissues of 12 representative paired HCC specimens are presented in Figure 8A. The correlation between TRs and BSSP4 was additionally analyzed. Linear regression analysis revealed a significant positive correlation between BSSP4 expression and TR levels, based on the T/N ratio (Spearman correlation coefficient = 0.445; 95% confidence interval (CI) = 0.154-0.665; P = 0.003) (Figure 8B). We have examined the correlation between BSSP4 and several parameters. A mild association between BSSP4 expression and distant metastasis is observed. Patients with T/N < 1.5 are less likely to have distant metastasis > 1 year than patients with T/N > 1.5 (P = 0.043). BSSP4 and VEGF levels in HCC tissues were determined using the Q-RT-PCR. We observed overexpression of BSSP4 and VEGF in 50% (26 of 52) and 61.5% (32 of 52) clinical specimens of HCC, respectively. Additionally, the T/N ratios of VEGF and BSSP4 expression revealed a significant positive correlation (Spearman correlation coefficient = 0.504; 95% confidence interval (CI) = 0.260-0.687; P = 0.0001) (Figure 8C; Table 1). We have added statistics information in “Additional file 6: Figure S6” to show the correlation in BSSP4 expression and important parameters in HCC patients. Among them, bilirubin level was significantly (*P* = 0.043), cirrhosis was marginally (*P* = 0.057) associated with BSSP4 expression. Notably, the expression of BSSP4 between TRα (Additional file 7: Figure S7A, Spearman r = 0.63, P < 0.001) and TRβ (Additional file 7: Figure S7B, Spearman r = 0.6, P < 0.001) were demonstrated a significantly positive correlation in public Oncomine microarray data sets. Further, Kaplan-Meier analysis was used to analyze the correlation of BSSP4 expression with clinical parameters, including tumor size, tumor grade, tumor number, microvascular invasion and macrovascular invasion…etc. (Additional file 8: Figure S8). Based on the data, the overall survival was significantly associated with HBsAg, ascites and tumor number. The HBsAg status was significantly associated with distant metastasis > 1y (*p* < 0.001). Moreover, the BSSP4 T/N ratio was also significatnly associated with distant metastasis > 1y (*p* = 0.043). The significantly associated parameters were shown in bold letters in the Additional file 8: Figure S8. Finally, the significant (*p* < 0.01) correlation between TR and BSSP4 was found in clinical HCC specimens (Figure 8A, B, C). Thus, the TR and BSSP4 expression levels were positively associated *in vitro* or *in vivo*. Both our *in vitro* and *in vivo* findings support the use of BSSP4 as an effective therapeutic target for HCC therapy.

**Table 1:** **The number of HCC patients stratified by TR**/**BSSP4**/**VEGF RNA expression levels**

**Target expression**	**TR**	**BSSP4**	**VEGF**
Low	6	18	7
Median	11	8	13
High	35	26	32

The expression levels (T/N) of TR, BSSP4 and VEGF were determined by QRT-PCR in HCC mRNA level (52 cases).

To determine the function of BSSP4 induced by T_3_, J7-TRα1 cells were employed. BSSP4 mRNA and protein expression was regulated in a dose- and time-dependent manner in J7-TRα1 cells (Figure 8D). To further investigate whether the T_3_-regulated effect *in vitro* is replicable *in vivo*, SCID mice were injected with J7-TRα1 cells and treated under several T_3_ conditions [5]. Mice injected with J7-TR cells displayed multiple macroscopic lung tumor nodules, as determined by hematoxylin and eosin (H & E) staining. Tumor size was calculated per cm^2^ of lung section and the metastatic index was significantly greater in hyperthyroid mice than in euthyroid condition. Higher T_3_ levels (hyperthyroid conditions) enhanced BSSP4 expression and number of lung foci, as observed with IHC (Figure 8E, upper panel) and H & E staining (Figure 8E, lower panel) [5]. The BSSP4 expression level in metastatic lung section foci of hyperthyroid was obviously higher than euthyroid mice. Moreover, we observed T_3_-induced cancer cell invasion and BSSP4 expression *in vivo*, suggesting that T_3_ influences tumor motility via BSSP4 regulation.

## Discussion

**Figure 9:**
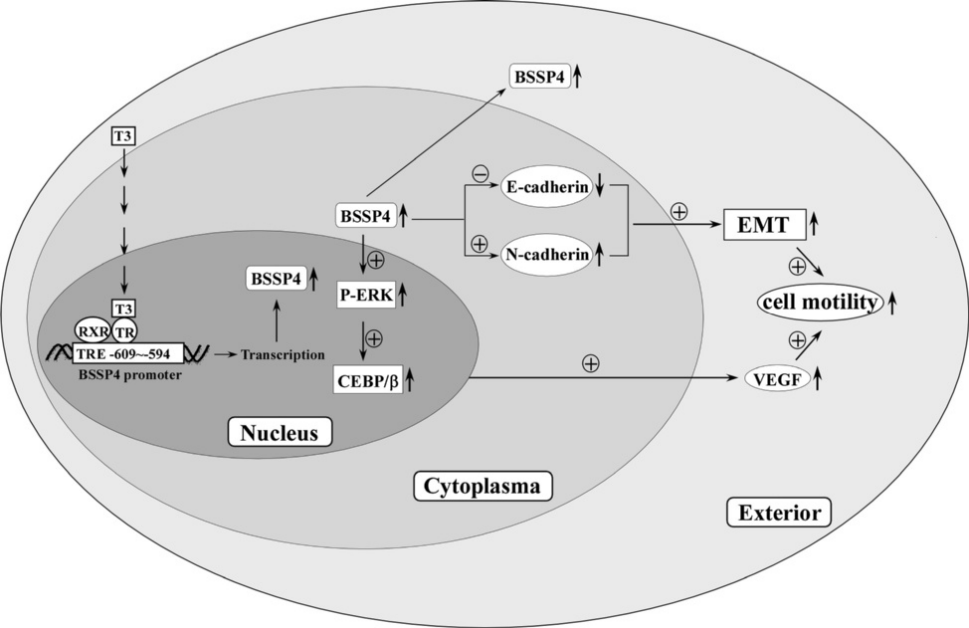
**Schematic representation of the pathway of TH**-**mediated cell motility.** Schematic representation of the pathway of TH-mediated cell motility. Addition of T_3_ (10 nM) to HepG2-TR α cells activates BSSP4 expression via direct binding of TR proteins to TRE (-609 ~ -594) of the BSSP4 promoter. P-ERK and C/EBPβ were upregulated in the nucleus, and VEGF was highly secreted in the medium of the BSSP4-overexpressed and T_3_-treated hepatoma cells. EMT-related proteins, such as E-cadherin and N-cadherin, were also influenced by BSSP4. BSSP4 and T_3_ exerted similar effects to promote cell motility via the EMT process and ERK-C/EBP β-VEGF cascade in hepatoma cells.

In a previous stable isotope labeling with amino acids in cell culture (SILAC)-based quantitative proteomics study of a thyroid hormone-regulated secretome in human hepatoma cells, BSSP4 was shown to be upregulated 3.25-fold by T_3_[5]. Here we have shown that BSSP4 is modulated by T_3_ at both the mRNA and protein levels. Additional studies confirmed that T_3_ regulates *BSSP4* at the transcriptional level, and TR and RXRα complexes directly bind TRE between positions -609 and -594 of the *BSSP4* gene 5΄-flanking region. Notably, cell lines overexpressing BSSP4 showed higher migration and invasion abilities, both *in vitro* and *in vivo*. Moreover, T_3_ appears to regulate BSSP4 via the ERK1/2/C/EBPβ/VEGF cascade, leading to cancer cell progression. The sequential events following *BSSP4* activation by T_3_ in hepatoma cells are illustrated in Figure 9.

BSSP4 was initially identified as a member of the serine protease family [7]. More recently, we identified several T_3_-modulated proteases, such as matrix metalloproteinases (MMPs) and cysteine cathepsins. MMPs are zinc- and calcium-dependent endopeptidases involved in proteolytic processing [21,22]. These proteases participate in cellular processes and intercellular communication during cancer progression and development [10,23,24]. Based on these findings, we hypothesized that the BSSP4 protease plays a role in T_3_-influenced cancer cell progression.

In cancer cell progression, epithelial-mesenchymal transition (EMT) is an important step leading to loss of cell-cell adhesion ability. E-cadherin is a transmembrane protein that participates in rearrangement of the cytoskeleton and cell-cell junctions [25]. E-cadherin overexpression attenuates tumor cell migration and metastasis [11,26]. When tumor cells from the epithelium transit to the mesenchymal type, N-cadherin expression is increased, and subsequently, cell conformation is altered. Consequently, migration and invasion abilities are increased after cells acquire N-cadherin expression [27]. Notably, expression of N-cadherin is increased and that of E-cadherin is decreased in BSSP4-overexpressing cells, compared with controls. We propose that BSSP4 accelerates tumor cell migration through the EMT or alterations in N-cadherin and E-cadherin expression.

VEGF is widely distributed in almost all malignant cancers, and considered an important tumor angiogenesis factor. The growth factor influences the vascular permeability of vascular endothelial cells and endothelial cell proliferation and migration *in vitro*, and generates new blood vessels *in vivo*. To date, several drugs have been effectively used to target VEGF with the aim of inhibiting tumor growth in a mouse model [28]. Chang *et al*. [29] reported that VEGF induces angiogenesis by distrusting the balance between proteases and protease inhibitors and causing basement membrane degradation. Highly expressed VEGF and increased microvessel density are correlated with decreased survival, and poor prognosis and progression in cancers [30,31]. Roudnicky *et al*. [32] demonstrated that VEGF is highly expressed in plasma of bladder cancer, compared with healthy controls, and promotes tumor cell migration via elevated expression of endothelial cell-specific molecule 1 (endocan). Yen *et al*. [33] reported that HBx induces cell proliferation and VEGF expression by upregulating the mTOR signaling pathway via IKK in HCC. BSSP4 and T_3_ enhanced VEGF secretion in the medium in both BSSP4-overexpressing and hepatoma cells in our experiments. Accordingly, we speculate that BSSP4 modulates cell motility through regulation of VEGF in the medium.

C/EBPβ is a transcription factor that regulates several target genes implicated in various processes, including cellular proliferation, migration, survival, metabolism and inflammation, and has diverse functions in several cancers [34]. Previously, Sebastian *et al*. [35] proposed that Ras-induced cell cycle arrest and tumor suppression occur via the ERK1/2 and C/EBPβ signaling pathways. Therefore, C/EBPβ may be effective as an activator of transcription factors and signal transducers, and modulate various target genes in response to hormone treatment [36]. Additionally, some groups have proposed that C/EBPβ is an effective regulator of oncogene-induced inflammatory secretome [37]. Consistent with earlier reports, we showed that BSSP4 increases C/EBPβ expression and cell motility through the ERK signaling pathway after U0126 treatment in both stable BSSP4-overexpressing and hepatoma cell lines.

Olazabal *et al*. [38] proposed that the pituitary hormone, prolactin, induces liver regeneration via activation of transcription factors involved in cell proliferation and liver-specific differentiation and metabolism, such as C/EBP and HNF families and the angiogenic and survival factors, VEGF and HIF-1α, after partial hepatectomy. Furthermore, Montano and co-workers reported that VEGF acts as a transcriptional target of HEXIM1, a transcription factor, tumor suppressor and cyclin-dependent kinase inhibitor, via regulation of C/EBP to influence cell invasion and myocardial proliferation and survival [39]. These data are consistent with our report that VEGF functions as an effector of the C/EBP pathway to influence cellular motility and may thus be employed as a downstream target.

Recently, the group of Clarke showed that a novel stroke therapy agent, perlecan domain V, promotes angiogenesis in brain endothelial cells, by inducing expression and secretion of VEGF via phosphorylation of the ERK1/2 pathway [16]. Shen *et al*. [40] showed that Notoginsenoside Ft1 (Ft1) stimulates angiogenesis by increasing VEGF expression and simultaneously activating the ERK signaling pathway. Feng and colleagues also proposed that low-power laser irradiation (LPL1) promotes VEGF expression and vascular endothelial cell proliferation via the ERK/Sp1 pathway [41]. In view of the several lines of evidence, including our results, we propose that VEGF may be a downstream signal and regulator mediating cell motility and angiogenesis.

Previously, Yasuda *et al*. [6] showed that BSSP4 is a protease catalyzing the conversion of pro-uPA to mature uPA, suggestive of a role in the fibrinolysis reaction. The fibrinolysis system is critical in the development of the tumor microenvironment and vascular diseases, such as fibrosis, fibrinolysis, thrombosis and atherosclerotic plaques [8]. Furthermore, we propose that the blood coagulation system is mediated by T_3_, based on MetaCore™ software analysis using the SILAC-based quantitative approach [5]. BSSP4 is abundantly found in endothelial cells, leading to the speculation that the protein is preferentially expressed in vascular cells [6]. The uPA system (uPA, tPA, uPAR, PAI-1 and BSSP4) is significantly linked with tumor cell metastasis, which is frequently associated with significant mortality and poor prognosis [5,8]. The uPA/uPAR system is highly expressed in almost all human cancers, and associated with short survival and high metastatic potency [8]. Additionally, pro-uPA has been identified as a substrate of BSSP4, which induces enhanced migration ability of pro-uPA-expressing smooth cells via basement membrane degradation [6]. In our experiments, BSSP4 enhanced migration and invasion abilities in HCC cell lines, both *in vitro* and *in vivo*, similar to that observed by other groups. In conclusion, T_3_-regulated BSSP4 may play a role in vascular endothelial cell motility through activation of VEGF to promote cancer cell progression.

## Conclusion

Our findings collectively support a potential role of T_3_ in cancer cell progression through regulation of the BSSP4 protease via the ERK 1/2-C/EBPβ-VEGF cascade. BSSP4 was overexpressed in clinical hepatocellular carcinoma (HCC) patients, compared with normal subjects, and positively associated with TRα1 and VEGF to a significant extent. Importantly, a mild association between BSSP4 expression and distant metastasis was observed. BSSP4 may thus be effectively utilized as a novel marker and anti-cancer therapeutic target in HCC.

## Materials and methods

### Cell culture

Human hepatoma cells, HepG2, Huh7 and J7, were routinely cultured at 37°C in a humidified atmosphere of 95% air and 5% CO_2_ in Dulbecco’s modified Eagle’s medium (DMEM) supplemented with 10% fetal bovine serum (FBS). HepG2 and J7 cell lines were stably transfected with TRα1 (HepG2-TRα1#1, HepG2-TRα1#2 and J7-TRα1) or TRβ1 (HepG2-TRβ1). The vector control cell line employed was HepG2-neo [42,43]. Huh7-BSSP4 and J7-BSSP4 represent the Huh7 and J7 cell lines expressing BSSP4, respectively. Serum was depleted of T_3_ (Td), as described previously [44].

### Preparation of conditioned medium

HepG2-TRα1#1, HepG2-TRα1#2, HepG2-TRβ1, HepG2-neo, J7-TRα1, Huh7-BSSP4 and J7-BSSP4 cells were grown to confluence in 10 cm cell culture dishes. Cells contacting dishes were washed twice with PBS, and subsequently incubated in serum-free medium and either treated with T_3_ for 24 h or left untreated. At the end of the treatment period, conditioned medium (CM) was collected and concentrated using spin columns with a molecular mass cut-off of 3 kDa (Amicon Ultra, Millipore, Billerica, MA).

### Immunoblot analysis

Total cell lysates and conditioned media were prepared, and protein concentrations determined with the Bradford assay kit (Pierce Biotechnology, Rockford, IL). Equivalent amounts of proteins were fractionated on a 10% sodium dodecyl sulfate (SDS)-polyacrylamide gel. Separated proteins were transferred on to a nitrocellulose membrane (pH 7.9, Amersham Biosciences Inc., Piscataway, NJ), blocked with 5% non-fat powdered milk, incubated with specific primary antibodies at 4°C overnight, and subsequently hybridized with the respective secondary antibody (HRP-conjugated mouse/rabbit/goat anti-IgG) for 1 h at room temperature. Finally, immune complexes were visualized using the chemiluminescence method with an ECL detection kit (Amersham) on Fuji X-ray film, as described previously [45].

### Quantitative reverse transcription polymerase chain reaction (Q-RT-PCR)

Total RNA was extracted from four T_3_-treated HepG2 isogenic cell lines using TRIzol reagent, as described previously [46]. Subsequently, cDNA was synthesized via RT-PCR with the Superscript II kit (Life Technologies, Karlsruhe, Germany). Real-time Q-RT-PCR was performed on a 15 μl reaction mixture containing 750 nM forward and reverse primers, varying amounts of template and 1 × SYBR Green reaction mix (Applied Biosystems, Foster City, CA). SYBR Green fluorescence was determined using the ABI PRISM 7500 detection system (Applied Biosystems). Primers were designed using Primer Express Software (Applied Biosystems). Genes were normalized against the ribosomal binding protein (*RiboL35A*) gene. The human BSSP4 oligonucleotides used in this study include the forward primer, 5′-GGTCCCAGAAGGTGGGTGTT -3, and reverse primer, 5′- ACGCACCAGGGCAATGTC -3′.

### Cloning and activities of *BSSP4* promoter fragments

Fragments of the *BSSP4* promoter (positions -2066 to -7) were ligated into the pA3TK vector (Promega Corp., Madison, WI), based on the published sequence. Several serial deletion and mutant constructs of the *BSSP4* promoter were amplified via PCR and cloned into pA3TK. Promoter sequences were confirmed using automated DNA sequencing. HepG2-TRα1 cells treated with 10 nM T_3_ for 24 h were cotransfected with 0.6 μg DNA/well of pA3TK vector containing the *BSSP4* promoter sequence and 0.3 μg of SVβ plasmid, a β-galactosidase expression vector (Clontech, Palo Alto, CA), in 24-well plates using TurboFect *in vitro* transfection reagent (Fermentas, Glen Burnie, MD) to determine the transcriptional activities of TREs within the *BSSP4* promoter. At the end of the treatment period, transfected and non-transfected cells were lysed, and luciferase and β-galactosidase activities measured. Luciferase activity was normalized to that of β-galactosidase, as described earlier [47].

### Chromatin immunoprecipitation (ChIP) assay

ChIP assays were performed to examine the interactions between TR and TRE on the *BSSP4* promoter [45]. HepG2-TRα1 cells treated with 10 nM T_3_ for 24 h or left untreated were harvested and cross-linked with 1% formaldehyde for 10 min at room temperature in DMEM medium. Reactions were terminated by adding 0.125 M glycine. Subsequently, cell lysates were washed three times with PBS, and resuspended in lysis buffer (150 mM NaCl, 5 mM EDTA, 50 mM Tris (pH 8.0), 0.1% SDS and 0.1% sodium deoxycholate) containing three protease inhibitors (1 mM PMSF, aprotinin, and leupeptin). Cell lysates were sonicated with a Misonix Sonicator 3000 Homogenizer (Mandel Scientific Company Inc., Guelph, ON, Canada) to disrupt chromatin. Sonicated DNA was between 200 and 1000 bp in length. Products were precleared with 60 μl protein A/G agarose (Sigma Chemicals, St. Louis, MO) for 2 h at 4°C. Complexes were immunoprecipitated with anti-TR (kindly provided by the laboratory of Dr. S-Y Cheng at the National Cancer Institute), anti-RXRα (Santa Cruz Biotechnology, Santa Cruz, CA) and anti-IgG antibodies (R & D Systems, Inc., Minneapolis, MN). The 100 bp fragment of the *BSSP4* promoter containing the predicted TRE region was amplified via PCR with the forward primer, 5′- CTCCAGGAACGACAGGAGGGCG - 3′, and reverse primer, 5′-GCCTGGGTTTGGAGAGGCTGAAGTC- 3′.

### Proliferation assay

Cells (4 × 10^4^) were seeded on a 6 cm dish and harvested at 1–7 days. The total number of cells in each condition provided a cell growth index via cell counting. Values are presented as fold increase in Huh7-BSSP4 and J7-BSSP4, relative to Huh7 and J7 control cells. Differences were analyzed using one-way ANOVA.

### Cloning of BSSP4

Total RNA (1 μg) was reverse-transcribed using Superscript II reverse transcriptase (Invitrogen) and Oligo (dT) to synthesize template cDNA. BSSP4 cDNA was amplified via PCR with the forward primer, 5S - CCAAGCTTATGGTGGTTTCTGGAGCGCCC-3′, and reverse primer, 5′ - CCGGAATTCCTAGGAGCGCGCGGCGG -3′, for 30 cycles at 95°C for 1 min, 60°C for 1 min and 72°C for 2 min. The BSSP4 open reading frame was ligated into pcDNA 3.0 expression vector, and the resulting construct sequenced to confirm the presence of the gene.

### Establishing Huh7 and J7 cell lines stably overexpressing BSSP4

The Huh7 cell line was transfected with the BSSP4 cDNA construct in 10 cm cell culture dishes using Lipofectamine Reagent (Invitrogen). After 24 h, transfected cells were transferred to medium containing G418 (400 μg/ml) for selection until the generation of a single cell clone. Expression of BSSP4 in Huh7 and J7 cells was detected using Western blotting.

### Effect of knocked-down BSSP4 expression

Short hairpin RNA clones targeting BSSP4 were purchased from the National RNAi Core Facility (Institute of Molecular Biology, Academia Sinica, Taiwan). Transfection of shRNA to against the endogenous BSSP4 gene in Mahlavu and J7-TR cells was transit performed using the Turbofect reagent (Invitrogen). The repression of the BSSP4 was confirmed by western blot analysis.

### *In vitro* migration and invasion assays

The influence of BSSP4 on migration and invasion abilities of Huh7-BSSP4 and J7-BSSP4 cells was determined with a rapid *in vitro* assay (Transwell) (Falcon BD, Franklin Lakes, New Jersey), as described previously [48]. Briefly, cell density was adjusted to 10^5^ cells/ml, and 100 μl of the suspension seeded on either non-matrigel-coated (migration) or matrigel-coated (invasion) (Becton-Dickinson) upper chambers of the Transwell plate. For both assays, the pore size of the upper chamber was 8 mm. The medium in the upper chamber was serum-free DMEM, while the lower chamber contained DMEM supplemented with 20% fetal bovine serum (FBS). After incubation for 24 h at 37°C, cells traversing the filter from the upper to lower chamber were examined via crystal violet staining and cell counting. Experiments were performed at least three times.

### Immunohistochemistry

Formalin-fixed and paraffin-embedded tissues from the lung of SCID mice were evaluated by hematoxylin and eosin (H & E) staining and immunohistochemistry using polyclonal antibody against BSSP4 (GeneTex, Inc, San Antonio, Texas) after the avidin-biotin complex method, as described previously. The positive staining consisted of cancer cells with dark brown of BSSP4 immunoreactivity.

### Animals

Similar conditions were employed with SCID mice containing various T_3_ levels (Group A to B) induced via injection of J7-TR cells [5]. Mice were divided into two groups, specifically, Group A (euthyroid) comprising control mice given normal drinking water, and Group B (hyperthyroid) administered drinking water augmented with T_3_ (2 mg/L) (Sigma Chem. Co., St. Louis, MO) after inoculation of tumor cells. Mice were sacrificed after about 1 month injection, livers and lungs were removed for tumor biopsy, and the T_3_ and TSH levels determined. The T_3_ and TSH levels in the serum of group A were 45.5 ng/dl and 0.246 mIU/ml, and in group B were 619 ng/dl and 0.008 mIU/ml, respectively. Tumor volume was calculated using the following equation: length × height × width. Formalin-fixed and paraffin-embedded tissues from SCID mice were evaluated by hematoxylin and eosin (H & E) staining and immunohistochemistry using polyclonal antibody against BSSP4 (GeneTex). All procedures were performed under sterile conditions in a laminar flow hood. Animal experiments were performed in accordance with United States National Institutes of Health guidelines and Chang-Gung Institutional Animal Care and Use Committee Guide for the Care and Use of Laboratory Animals.

### Human HCC specimens

With informed consent, patients with HCC diagnosed between 2000 and 2003 were selected consecutively for this study. All samples of HCC tissues with paired adjacent normal liver tissues were obtained during surgical resection from the Chang Gung Memorial Hospital medical research center for western blot and Q-RT-PCR analysis. The study protocol was approved by the Medical Ethics and Human Clinical Trial Committee at Chang-Gung Memorial Hospital.

### Statistical analysis

Data are expressed as mean values ± SEM of at least three experiments. Statistical analysis was performed using the Student’s *t* test and one-way ANOVA analysis. *P* < 0.05 was considered statistically significant.

## Abbreviations

BSSP4: Brain-specific serine protease 4; C/EBPβ: CCAAT/enhancer-binding protein β; ERK: Extracellular signal-regulated kinase; HCC: Hepatocellular carcinoma; MAPK: Mitogen-activated protein kinase; TH: Thyroid hormone; TREs: Thyroid hormone response elements; uPA: urokinase-type plasminogen activator; VEGF: Vascular endothelial growth factor.

## Competing interest

The authors declare that they have no competing interests.

## Authors’ contribution

Conception and design: C.Y. Chen, I. H Chung, Y.H. Lin, H.C. Chi, K.H. Lin Development of methodology: Y.H. Lin, C.Y. Chen, M. M. Tsai, K.H. Lin Acquisition of data (provided animals, acquired and managed patients, provided facilities, etc.): C.Y. Chen, I. H Chung, Y. C. Wang , Y.H. Tseng. Analysis and interpretation of data (e.g., statistical analysis, biostatistics, computational analysis): C.Y. Chen, I. H Chung, Y.H. Tseng, C.Y. Tsai, C.P. Chen, T. I. Wu, C. T. Yeh, D. I. Tai, K.H. Lin. Writing, review, and/or revision of the manuscript: C.Y. Chen, I. H Chung, K.H. Lin Administrative, technical, or material support (i.e., reporting or orga-nizing data, constructing databases): C.Y. Tsai, H.C. Chi, K.H. Lin Study supervision: C.Y. Chen, I. H Chung, K.H. Lin. All authors read and approved the final manuscript.

## Supplementary Material

Additional file 1: Figure S1 Effect of TR on T_3_ induction of BSSP4 expression in SK-Hep-1 cell. The TRα and TRβ expression were depleted with siRNA in SK-Hep1 cell (A). The BSSP4 protein level was examined in the conditioned medium of SK-Hep-1 and SK-Hep-1-siTRαβ cells treated with 1 or 10nM T3 (24 h and 48 h) and analyzing by Western blotting (B). Click here for file

Additional file 2: Figure S2 Expression of BSSP4 in hepatoma cell lines. The BSSP4, or TRα/TRβ expression levels were detected in six available hepatoma cells (SK-Hep-1, Mahlavu, Huh7, HepG2, J7 and Hep-3B) analyzing by Western blotting. Click here for file

Additional file 3: Figure S3 Effect of proliferation ability by BSSP4 in hepatoma cell lines. Cell growth rates were determined from 1 to 7 days, and expressed as the total number of cells representing index of proliferation ability. (A) Huh7, (B) J7. Click here for file

Additional file 4: Figure S4 Regulation of VEGFR by BSSP4 and T3 in hepatoma cells. The p-VEGFR and VEGFR expression were examined in Huh7 BSSP4-overexpressing (A) and T3-treated HepG2-TRα1 (B) Cells by Western blotting. Click here for file

Additional file 5: Figure S5 Pathways or molecules regulated by BSSP4 in hepatoma cells. Several categories based on the functions such as (A) cytokines and chemokine pathway (IL-18, CCL7, CXCL12), (B) cell to cell adhesion pathway (PNN, SYK, MCAM), (C) metastasis-related genes (GNRH1, TIMP2, KISS1R, TSHR, TRPM1, SSTR2) (D) Transcription factors and regulators (RORB, NR4A3, SMAD2, SMAD4) (E) ECM cleavage pathway (MMP7) and (F) cell cycle regulation (PTEN) were determined by metastasis-associated PCR array in Huh7 BSSP4-overexpressing stable cells. Click here for file

Additional file 6: Figure S6 Clinicopathological correlation of BSSP4 expression and several parameters in hepatoma patients. Click here for file

Additional file 7: Figure S7 Positive correlation of BSSP4 and TRα/TRβ expression levels. The correlation between BSSP4 and TRα (A) and TRβ (B) were analyzed from Oncomine microarray data sets [1]. Click here for file

Additional file 8: Figure S8 Clinicopathological correlation of BSSP4 expression and overall survival rate in hepatoma patients. Click here for file
