# Supplementary figures and images for: Thyroid hormone enhanced human hepatoma cell motility involves brain-specific serine protease 4 activation via ERK signaling
Source: Mol Cancer. 2014 Jul 1;13:162. doi: 10.1186/1476-4598-13-162 (PMC4087245; doi:10.1186/1476-4598-13-162)

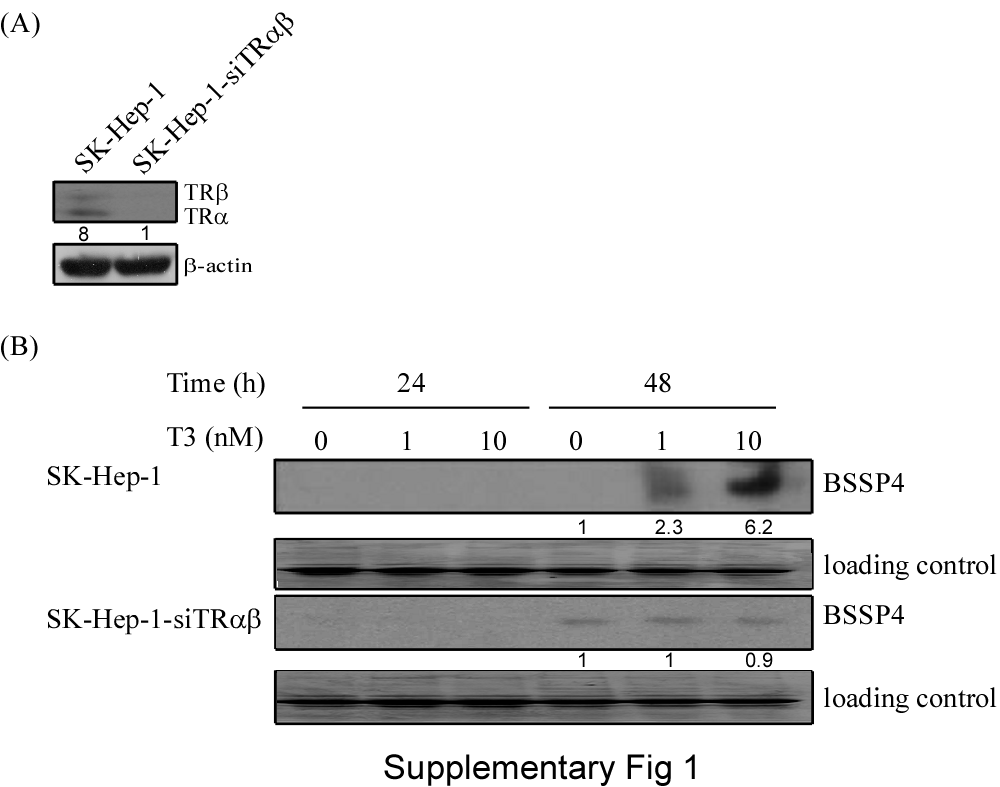

Supplement: Additional file 1: Figure S1 — Effect of TR on T3 induction of BSSP4 expression in SK-Hep-1 cell. The TRα and TRβ expression were depleted with siRNA in SK-Hep1 cell (A). The BSSP4 protein level was examined in the conditioned medium of SK-Hep-1 and SK-Hep-1-siTRαβ cells treated with 1 or 10nM T3 (24 h and 48 h) and analyzing by Western blotting (B). [file 1476-4598-13-162-S1.tiff]

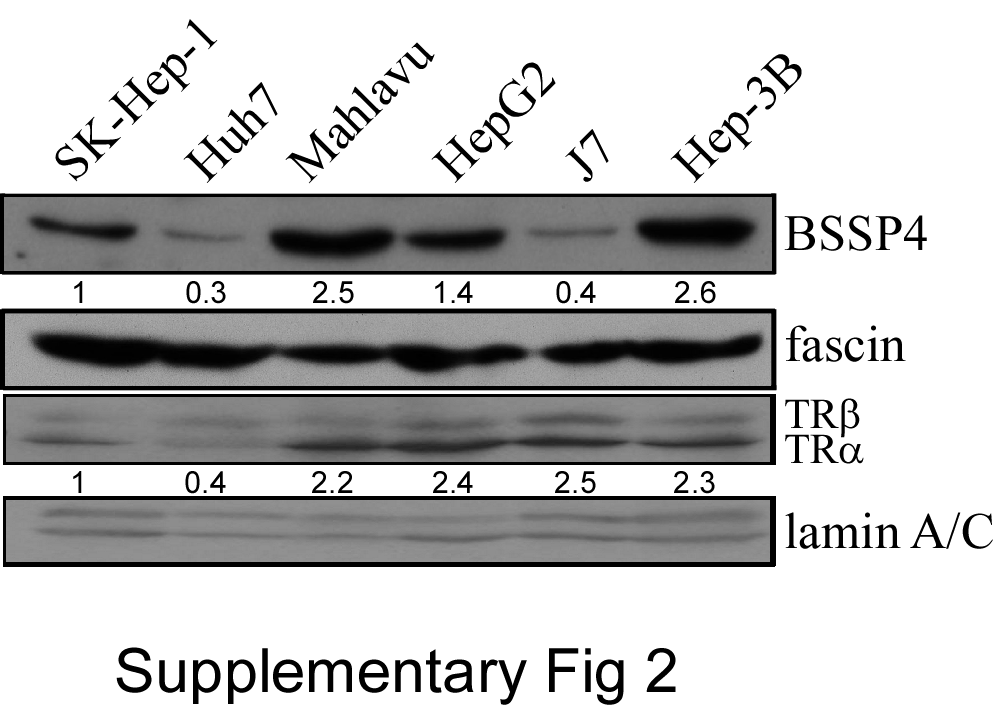

Supplement: Additional file 2: Figure S2 — Expression of BSSP4 in hepatoma cell lines. The BSSP4, or TRα/TRβ expression levels were detected in six available hepatoma cells (SK-Hep-1, Mahlavu, Huh7, HepG2, J7 and Hep-3B) analyzing by Western blotting. [file 1476-4598-13-162-S2.tiff]

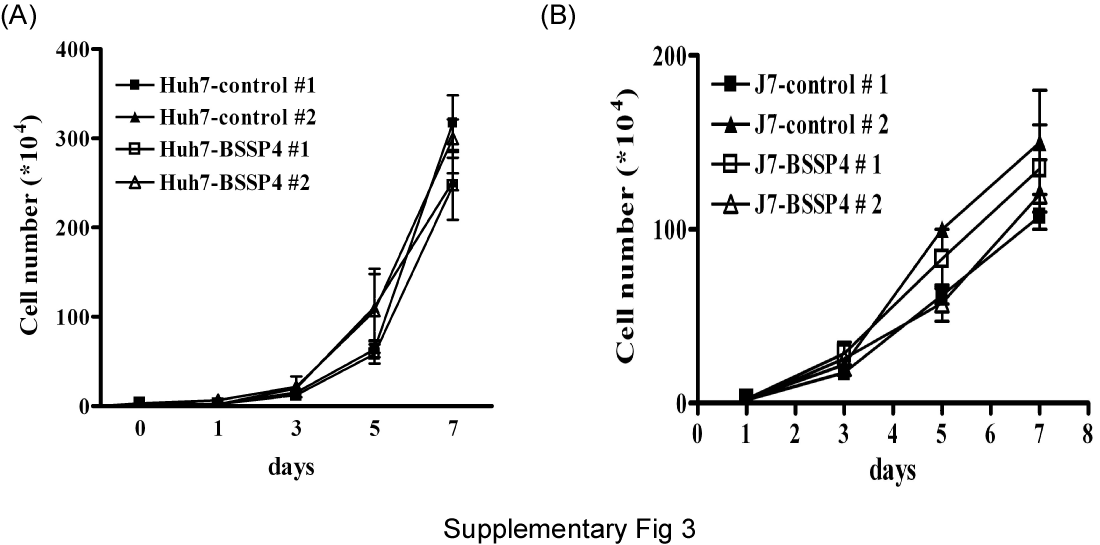

Supplement: Additional file 3: Figure S3 — Effect of proliferation ability by BSSP4 in hepatoma cell lines. Cell growth rates were determined from 1 to 7 days, and expressed as the total number of cells representing index of proliferation ability. (A) Huh7, (B) J7. [file 1476-4598-13-162-S3.tiff]

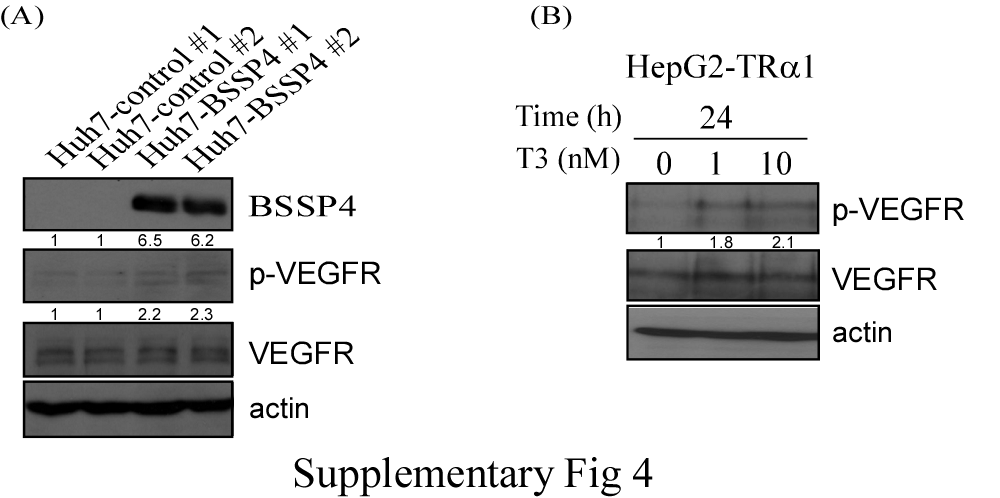

Supplement: Additional file 4: Figure S4 — Regulation of VEGFR by BSSP4 and T3 in hepatoma cells. The p-VEGFR and VEGFR expression were examined in Huh7 BSSP4-overexpressing (A) and T3-treated HepG2-TRα1 (B) Cells by Western blotting. [file 1476-4598-13-162-S4.tiff]

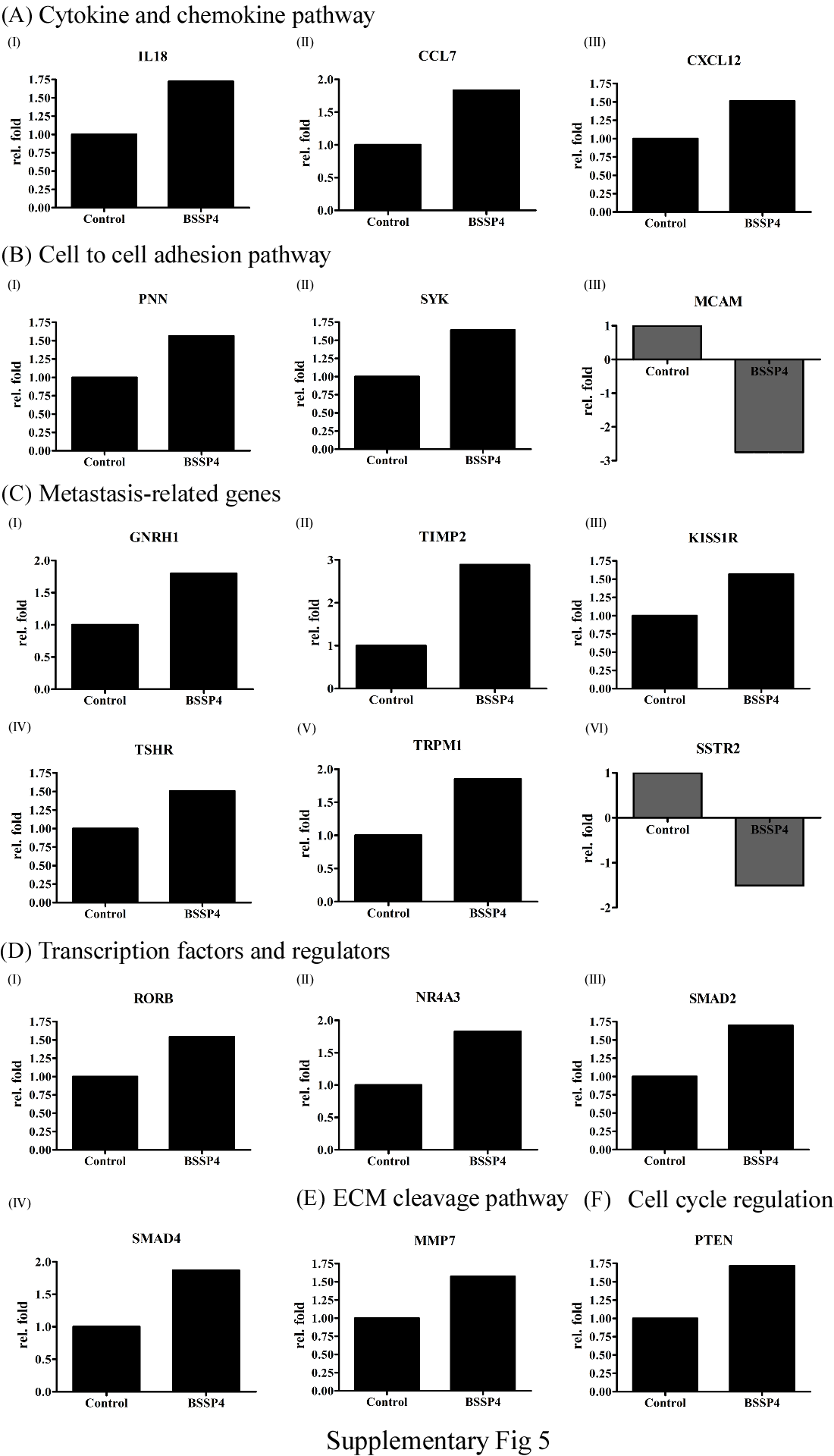

Supplement: Additional file 5: Figure S5 — Pathways or molecules regulated by BSSP4 in hepatoma cells. Several categories based on the functions such as (A) cytokines and chemokine pathway (IL-18, CCL7, CXCL12), (B) cell to cell adhesion pathway (PNN, SYK, MCAM), (C) metastasis-related genes (GNRH1, TIMP2, KISS1R, TSHR, TRPM1, SSTR2) (D) Transcription factors and regulators (RORB, NR4A3, SMAD2, SMAD4) (E) ECM cleavage pathway (MMP7) and (F) cell cycle regulation (PTEN) were determined by metastasis-associated PCR array in Huh7 BSSP4-overexpressing stable cells. [file 1476-4598-13-162-S5.tiff]

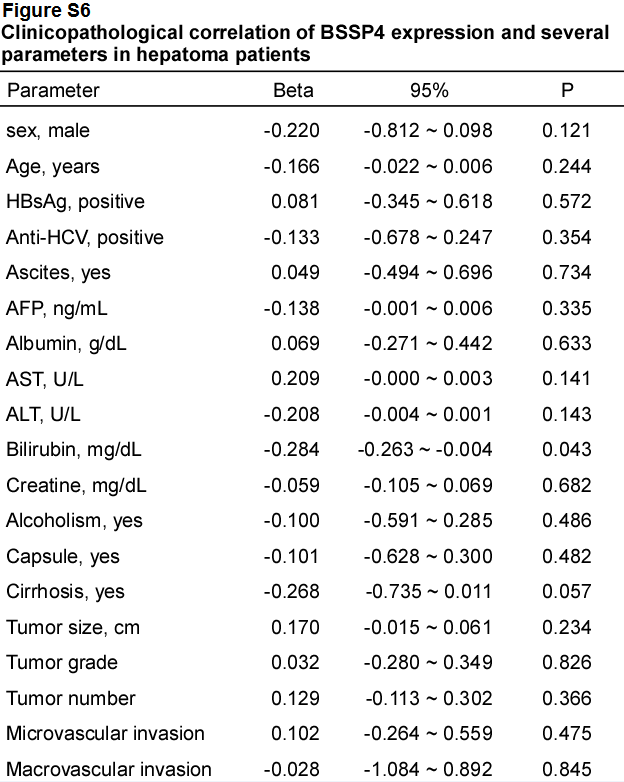

Supplement: Additional file 6: Figure S6 — Clinicopathological correlation of BSSP4 expression and several parameters in hepatoma patients. [file 1476-4598-13-162-S6.tiff]

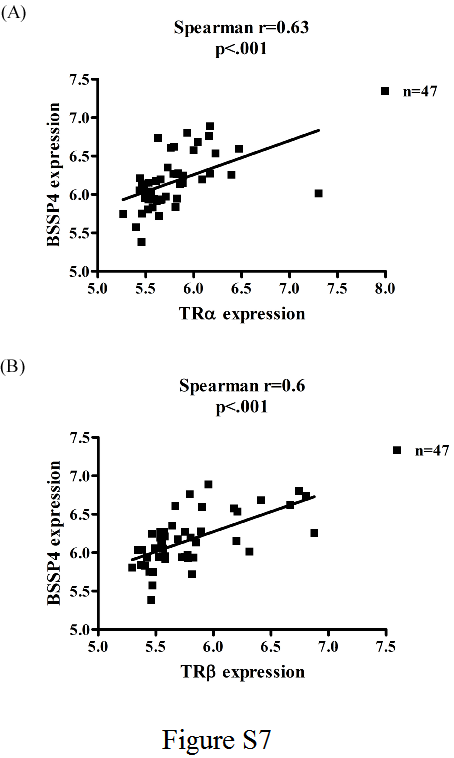

Supplement: Additional file 7: Figure S7 — Positive correlation of BSSP4 and TRα/TRβ expression levels. The correlation between BSSP4 and TRα (A) and TRβ (B) were analyzed from Oncomine microarray data sets [1]. [file 1476-4598-13-162-S7.tiff]

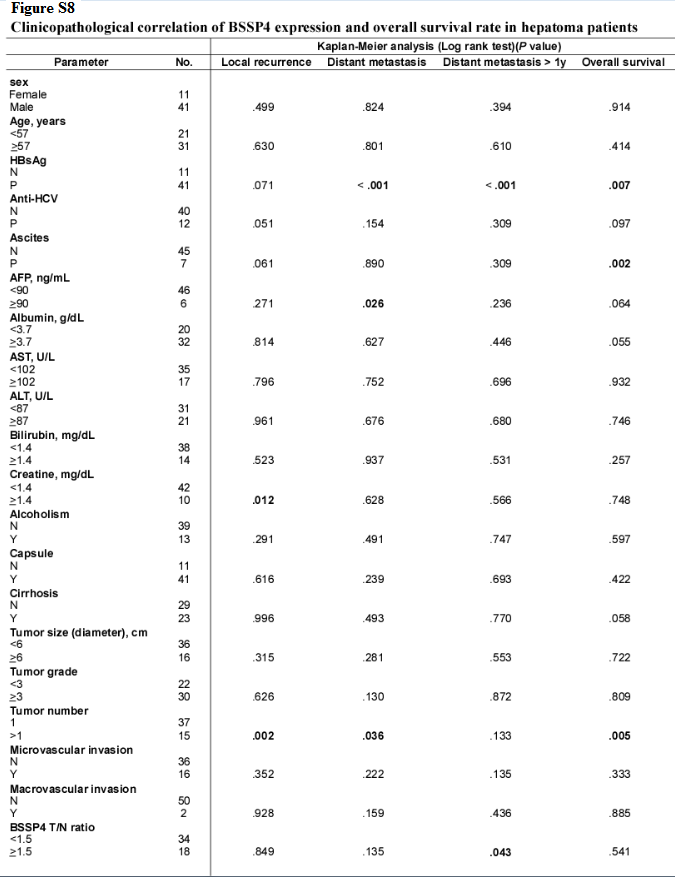

Supplement: Additional file 8: Figure S8 — Clinicopathological correlation of BSSP4 expression and overall survival rate in hepatoma patients. [file 1476-4598-13-162-S8.tiff]
